# Preoperative contrast-enhanced computed tomographic characterisation of pancreatic cystic lesions: A prospective study

**DOI:** 10.4102/sajr.v23i1.1727

**Published:** 2019-06-10

**Authors:** Dar M. Saleem, Wani A. Haseeb, Arshed H. Parry, Robbani Irfan, Najar M. Muzaffar, Gojwari Tariq, Shah O. Javed, Imza Feroz

**Affiliations:** 1 Department of Radiodiagnosis, Sher-I-Kashmir Institute of Medical Sciences, Srinagar, India; 2 Department of Surgical Gastroenterology, Sher-I-Kashmir Institute of Medical Sciences, Srinagar, India

**Keywords:** Serous cystadenoma, mucinous cystadenoma, solid pseudo-papillary tumour, intra-ductal papillary mucinous neoplasm, simple pancreatic cyst, pancreatic lymphangioma

## Abstract

**Background:**

Characterisation of pancreatic cystic lesions has a direct role in their management and computed tomography is the mainstay of investigation for diagnosing and characterising them.

**Objectives:**

The aim of this study was to prospectively assess the diagnostic accuracy of contrast-enhanced computed tomography (CECT) in preoperative characterisation of pancreatic cystic lesions with histopathology as the reference standard.

**Method:**

A total of 38 patients with cystic pancreatic lesions diagnosed after clinical, laboratory and sonographic evaluation, irrespective of age, were preoperatively evaluated with CECT. Images were reviewed for the general characteristics of the lesions on pre-contrast and portal venous phase images and overall diagnostic accuracy calculated. Imaging findings were compared with histopathology, or cytology and/or intra-operative findings.

**Results:**

Serous cystadenoma (SCA) was the most common cystic pancreatic lesion found in 31.6% of patients followed by mucinous cystadenoma (MCA) (26.3%), solid pseudo-papillary tumour (SPT) (21.1%) and intra-ductal papillary mucinous neoplasm (IPMN) (10.5%). Three patients (7.9%) had simple cysts and one patient (2.6%) had a lymphangioma. The diagnostic accuracy of CECT for pancreatic cystic lesions was found to be 72.5%

**Conclusion:**

The diagnostic accuracy of computed tomography (CT) was high for SCA, IPMN and pancreatic cysts, and low for MCA and SPT. Combination of a multiloculated cystic lesion with locule size of less than 20 mm, septal enhancement with relative lack of wall enhancement, central scar and lobulated outline are highly specific for SCA. Unilocular or macro-cystic pattern with locule size of more than 20 mm, female gender and wall enhancement with smooth external contour are pointers towards MCA. Solid cystic pancreatic head lesions in young females may be suggestive of SPT. A dilated main pancreatic duct in a cystic lesion with internal septations may point towards IPMN. Fluid attenuation lesions with imperceptible non-enhancing wall indicate pancreatic cysts. Lastly, pseudocysts and neuroendocrine tumours with cystic components are great mimickers of pancreatic cystic lesions, and a history of pancreatitis and hormonal profile of patients should always be sought.

## Introduction

Pancreatic lesions can be either solid, cystic or solid–cystic in nature, either of which can be benign, borderline or malignant. Most cystic lesions of the pancreas are benign.^1,2,3^ Characterisation becomes the *sine qua non* in distinguishing pancreatic cystic lesions as it has a direct bearing on their management, for example, to differentiate true cystic neoplasms from pancreatic pseudocysts. Serous cystadenomas (SCAs), mucinous cystic neoplasms and intra-ductal papillary mucinous neoplasms constitute more than 90% of primary cystic pancreatic neoplasms.^2^ Simple cysts and SCAs are benign and, if asymptomatic, can be safely followed. On the contrary, mucinous neoplasms are potentially malignant, justifying their surgical resection.^1,2,3^ Cystic pancreatic lesions are usually found incidentally on imaging studies performed for other reasons, and as many as 35% of patients are totally asymptomatic at the time of diagnosis.^1,2,3^

Similar to Bosniak’s classification for renal cysts, a radiological classification based on imaging features of pancreatic cystic lesions has been suggested.^4^ Pancreatic cystic lesions include unilocular cysts, micro-cystic lesions, macro-cystic lesions and mixed cystic lesions with a solid component. Fine needle aspiration provides a tissue diagnosis, but is often non-diagnostic because of sampling error^5^ and various limitations.^6^ Its sensitivity for diagnosis of cystic lesions is much lower than for solid lesions.^7,8^ Imaging modalities such as ultrasonography (US), computed tomography (CT), magnetic resonance imaging (MRI), positron emission tomography and endoscopic US play a key role in characterisation, staging, surgical planning and assessment for treatment.^9,10,11^ The best approach to obtain an exact preoperative diagnosis is the combined evaluation of all available clinical, serological, radiological and biopsy findings.

Ultrasonography is not an ideal screening tool for the detection of pancreatic masses because of its relatively low sensitivity.^12,13^ The major limitation of endoscopic US is its inability to stage disease beyond the pancreas, thus it is generally used as an adjunct to or after multidetector computed tomography (MDCT). Magnetic resonance imaging is useful for iso-enhancing pancreatic masses that are not directly seen on CT.^14^ Susceptibility of MRI to significant image degradation by respiratory motion artefact is even more so in contrast studies, which is often critical for characterising pancreatic lesions, limits its diagnostic capability.^15,16^ In spite of advances in MRI in abdominal imaging, CT is still the preferred imaging modality for both initial detection and characterisation of cystic pancreatic lesions, especially the macro-cystic ones.^17^ Computed tomography is an excellent imaging modality for pancreatic cystic lesions because of its widespread availability and ability to detect cysts.^18^

This study was undertaken to evaluate the possible role of CT in the preoperative characterisation of pancreatic lesions, on the basis of various morphological characteristics, because preoperative fine needle aspiration cytology may lack the desired diagnostic accuracy, more so in cases of cystic lesions.

## Materials and methods

Prior to imaging, the risks and benefits were discussed with each patient and informed consent taken. Patients with cystic pancreatic lesions diagnosed after clinical, laboratory and sonographic evaluation, irrespective of age, were included in this study. Patients with inflammatory, parasitic or purely solid pancreatic lesions based on imaging, clinical and laboratory profiles and patients with deranged renal function tests, contrast allergy and pregnancy were excluded from the study. To characterise the pancreatic cystic lesions, a real-time ultrasound examination of the abdomen was done, followed by contrast-enhanced CT (CECT).

Ultrasound was done using a 3.5 MHz curvilinear and 7.5 MHz linear array probe on an Aloka Prosound SSD-3500SX machine. All CT examinations were performed on a Siemens Somatom Sensation 64. Contrast-enhanced CT of the abdomen was performed on all patients. The anterior cubital vein of the patients was cannulated using an 18G intravenous cannula. A pressure injector was used to inject 120 mL–150 mL of contrast (omnipaque) as per body weight. After obtaining the topogram and non-contrast images of the abdomen, a portal venous phase CT was performed 40–60 s after the start of contrast injection. On a workstation, multi-planar coronal, sagittal and axial reconstructions were performed. Analysis of CT images was performed on a picture archiving and communication system (PACS) workstation monitor by an experienced radiologist with more than 15 years of abdominal imaging experience. The radiologist was blinded to the histopathological and surgical findings, but not to the clinical history of the patients. Images were reviewed for the general characteristics of the lesions on pre-contrast and portal venous phase images. Imaging findings were compared with histopathology/cytology and/or intra-operative findings. The final diagnosis was confirmed by histopathology/cytology in all cases.

## Statistical analysis

The data were analysed using statistical software SPSS v20 and STATA v11. Categorical variables were described in terms of frequency and percentage and the continuous variables in terms of descriptive statistics like mean, standard deviation (SD), minimum, maximum and range. Also, the sensitivity, specificity and accuracy were calculated. All the results were determined at the 5% significance level.

## Ethical consideration

This was a prospective observational study conducted between October 2015 and December 2018, with approval from the Institutional Ethical Committee (IEC) (No. SIMS 1 31/IEC-SKIMS/2015–75) and a final sample size of 38.

## Results

Of the 38 patients diagnosed with cystic pancreatic lesions on imaging, 12 patients (31.6%) had SCA, 10 patients (26.3%) had mucinous cystadenomas (MCA), 8 (21.1%) had solid pseudo-papillary tumours (SPT), 4 (10.5%) patients had intra-ductal papillary mucinous neoplasms (IPMN), 3 patients (7.9%) had simple cysts and 1 patient (2.6%) had a lymphangioma. The age group and mean age (in years) at the time of diagnosis and gender distribution of various pancreatic cystic lesions are shown in Table 1. Distribution with respect to anatomic location in the pancreas is shown in Table 2. The mean size of a SCA on CT was 3.5 cm and that of MCA was 7.3 cm; mean size of SPT was 5.3 cm.

**TABLE 1:** Age, mean age (in years) and gender distribution of various pancreatic cystic lesions at the time of diagnosis.

Final diagnosis	Age (years)						Mean age (years)	Gender	
	<30		30–50		>50			Male	Female
	*n*	%	*n*	%	*n*	%			
SCA	4	33.3	6	50	2	16.7	44.5	2	10
MCA	2	20	4	60	2	20	47.0	0	10
SPT	6	75	2	25	0	0	26.5	0	8
IPMN	0	0	2	50	2	50	56.0	4	0
Simple cyst	3	100	0	0	0	0	27.0	1	2
Lymphangioma	0	0	0	0	1	100	55.0	0	1

SCA, serous cystadenoma; MCA, mucinous cystadenoma; SPT, solid pseudo-papillary tumour; IPMN, intra-ductal papillary mucinous neoplasm.

**TABLE 2:** Distribution of pancreatic cystic lesions as per the anatomic site.

Diagnosis	Site of the lesion in pancreas					
	Head or uncinate		Body		Tail	
	*n*	%	*n*	%	*n*	%
SCA	8	66.7	4	33.3	0	0
MCA	0	0	2	20	8	80
SPT	6	75	0	0	2	25
IPMN	2	50	2	50	0	0
Simple cyst	0	0	3	100	0	0
Lymphangioma	0	0	1	100	0	0

SCA, serous cystadenoma; MCA, mucinous cystadenoma; SPT, solid pseudo-papillary tumour; IPMN, intra-ductal papillary mucinous neoplasm.

The enhancement pattern on CECT was defined as either absent or enhancement of the wall, septae, both septae and wall and solid–cystic with enhancement of solid component, as shown in Table 3. Excluding SPT that has a solid component, Table 4 shows the loculation pattern and the size of the locules (in multilocular cystic lesions) in the truly cystic pancreatic lesions. Intra-hepatic biliary radicles and common bile duct (CBD) were not dilated in any of the cystic lesions. Main pancreatic duct (MPD) dilatation was seen in the four patients (100%) with IPMN and in two patients (20%) with MCA; normal MPD seen in the rest of the cystic lesions.

**TABLE 3:** Enhancement pattern on computed tomography of various pancreatic cystic lesions.

Final diagnosis	Enhancement				
	Nil	Wall	Septal	Wall and septal	Solid cystic
SCA	2	0	10	0	0
MCA	0	6	0	4	0
SPT	0	0	0	0	8
IPMN	0	0	4	0	0
Simple cyst	3	0	0	0	0
Lymphangioma	0	0	1	0	0

SCA, serous cystadenoma; MCA, mucinous cystadenoma; SPT, solid pseudo-papillary tumour; IPMN, intra-ductal papillary mucinous neoplasm.

**TABLE 4:** Loculation pattern and size of pancreatic cystic lesions.

Lesion	Unilocular		>1 Locule		Size of locules	
	*n*	%	*n*	%	≤20 mm	>20 mm
SCA	2	16.7	10	83.3	10	0
MCA	6	60	4	40	0	4
IPMN	0	0	4	100	4	0
Simple cyst	3	100	0	0	0	0
Lymphangioma	0	0	1	100	1	0

SCA, serous cystadenoma; MCA, mucinous cystadenoma; SPT, solid pseudo-papillary tumour; IPMN, intra-ductal papillary mucinous neoplasm.

Ten of the 12 cystic lesions diagnosed as SCA on CT proved to be SCA on histopathology examination (HPE) as well; one was diagnosed as a pseudocyst of pancreas and the other as MCA on HPE. Six of the 10 patients were correctly diagnosed as MCA on CT; three were found to be pseudocysts and one was a SCA on HPE or surgery. Five of the eight patients were correctly diagnosed as SPT on CT, while the other three proved to be neuroendocrine tumours with cystic components on HPE. All of the four cases of IPMN, three cases of simple cysts and one case of lymphangioma were correctly identified on CT. The diagnostic accuracy of CECT for pancreatic cystic lesions was found to be 72.5%, with 57.16% and 83.89% as the lower and upper 95% confidence interval limits.

## Discussion

Serous cystadenoma was the most common cystic pancreatic lesion found in 31.6% of patients in our study followed by MCA (26.3%), SPT (21.1%) and IPMN (10.5%). Three patients (7.9%) had simple cysts and one patient (2.6%) had a lymphangioma.

**FIGURE 1:**
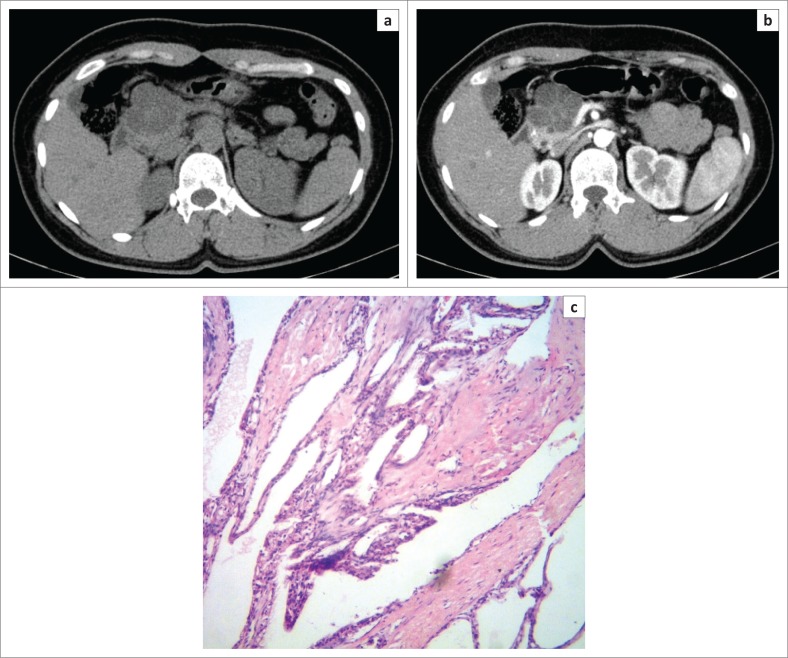
Axial non-contrast-enhanced computed tomography image (a) in a 37-year-old female patient showing fluid density lobulated lesion in the head of pancreas. Axial contrast-enhanced computed tomography image (b) in the same patient showing a multiloculated cystic mass in the head of pancreas with enhancing septations, a central stellate scar and no definite wall enhancement, suggestive of serous cystadenoma. High power magnification (40X) photomicrograph (c) of the same patient confirmed the diagnosis of micro-cystic serous cystadenoma.

Serous cystadenoma was found predominantly in females in our study (male:female ratio of 1:5) with mean age of 44.5 years, similar to the studies of Atalay et al.^19^ and Parra-Herran et al.^20^ Serous cystadenomas were predominantly located in the head or uncinate process (66.7%) (Figure 1) and the body of pancreas (33.3%). None were located in the pancreatic tail. Megibow et al.^21^ reported that serous cystic neoplasm is most often found in the pancreatic head. Atalay et al.^19^ reported 23 cases of SCAs of pancreas, all of which were present in the head and body of the pancreas. Of the 12 SCAs, 2 (16.7%) were unilocular and 10 (83.3%) were multiloculated, consistent with the study of Atalay et al.^19^ All the multiloculated SCAs had the size of the largest locule, less than 20 mm. Johnson et al.^22^ reported that on ultrasound, SCAs usually have more than six loculi that are less than 2 cm in diameter. Bhatt and Vaishnav^23^ found that SCAs were approximately 5 cm–6 cm in size with small internal cysts < 2cm, with septations. However, Curry et al.^24^ reported that the largest cyst in each tumour was smaller than 2 cm in only 14 (64%) out of the 22 patients. Four (33.3%) SCAs had a lobulated outline that is characteristic of SCA,^18,25^ while others had a regular outline. Central scar, a characteristic of SCA,^18,25^ was noted only in two (16.7%) of the SCAs (Figure 1b). Torresan et al.^26^ reported that although seen in less than 20% of SCAs, demonstration of a central scar by CT or MRI is a highly diagnostic feature of a SCA. Calcification was seen in none of the 12 SCAs in our study. However, central calcification can be seen in SCAs within the fibrous stroma.^25^ Curry^24^ reported central calcification in 10% of all cystic pancreatic lesions in their study (5/50), of which 80% were SCAs. The majority of SCAs in our study (10/12) showed septal enhancement (Figure 1b) on CECT, as also reported by Balthazar et al.^27^ Biliary radicles, CBD and MPD were not dilated in any SCAs in our study, which is consistent with the literature. Rarely, giant lesions can cause compression of the MPD or bile ducts.^28^ Histopathology examination revealed the two pancreatic cystic lesions presumptively diagnosed as SCAs on CT in our study to be pseudocyst of pancreas and MCA. Both these lesions were unilocular or oligocystic, and hence MCA and pseudocysts are close differentials.

**FIGURE 2:**
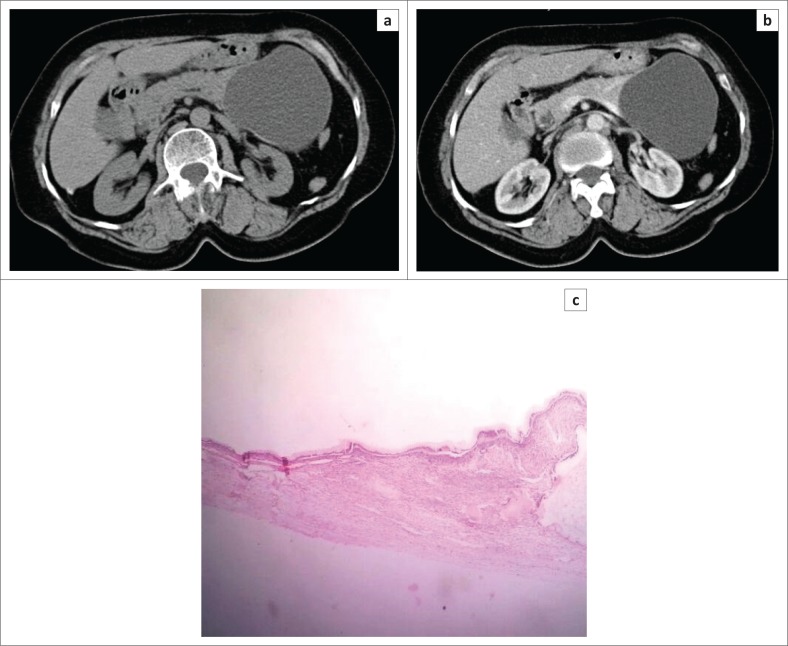
Axial non-contrast-enhanced computed tomography image (a) in a 49-year-old female patient showing large fluid attenuation cystic lesion in the tail of pancreas with a smooth external contour. Axial contrast-enhanced computed tomography image (b) in the same patient showing a non-enhancing cystic lesion in the tail of pancreas with subtle wall enhancement, suggestive of mucinous cystadenoma. Low power magnification (scanner 4X) photomicrograph (c) of the same patient confirmed the diagnosis of mucinous cystadenoma.

Mucinous cystadenoma was the second most common pancreatic cystic lesion (26.3%) with a mean age of 47 years at the time of diagnosis and seen exclusively in females in our study. They were predominantly located in tail (80%) (Figure 2) and pancreatic body (20%), with no lesions seen in head or uncinate process. Mean size of MCA (7.3 cm) was larger than SCA and SPT in our study. The findings are consistent with the majority of other studies^19,20,29^ The majority of MCAs were unilocular (60%) with 40% appearing multilocular in our study. The size of the largest locule was >20 mm in multiloculated lesions. Unilocular or macro-cystic pattern,^30^ with wall enhancement, is very helpful in diagnosing MCA on CT. Curry^24^ reported that the largest locule in each tumour was larger than 2 cm in 24 patients (86%) with MCA. Mucinous cystadenomas showed a smooth external contour (Figure 2) in our study consistent with the world literature.^31^ Calcifications were present in none of these lesions, although MCAs may have a peripheral eggshell calcification.^25^ Wall enhancement was seen in all cases of MCAs in our study on CECT (Figure 2b), with only 40% cases showing variable enhancement of septations. Cohen-Scali et al.^32^ reported that the lack of wall enhancement was specific for macro-cystic SCA in comparison with MCA. Biliary radicles, CBD and MPD were not dilated in any MCA; however, Warshaw et al.^31^ reported that very rarely they can cause ductal obstruction, but do not communicate with the MPD. Three cases were misdiagnosed as MCA on CT, which turned out to be pseudocysts. Pseudocyst with wall enhancement is a close differential for MCA on CT, especially if the past history of pancreatitis is not forthcoming. Because of significant wall enhancement, one unilocular SCA was wrongly labelled as MCA on CT.

**FIGURE 3:**
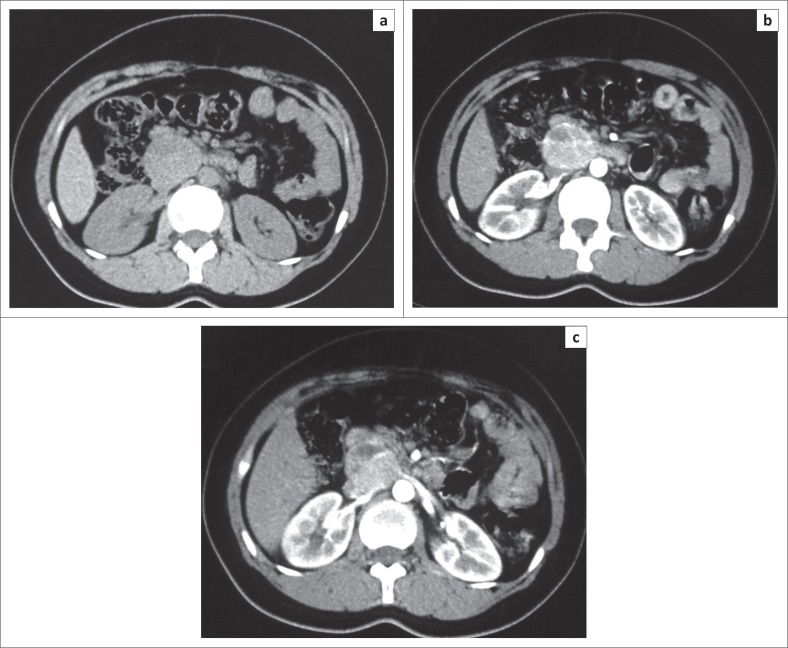
Axial non-contrast-enhanced computed tomography image (a) in a 26-year-old female patient showing a well-defined lesion in the pancreatic head or uncinate process with axial contrast-enhanced (b) and magnified post-contrast computed tomography image (c) showing an enhancing solid lesion with a non-enhancing component suggestive of solid pseudo-papillary tumour. Histopathology examination confirmed the same.

**FIGURE 4:**
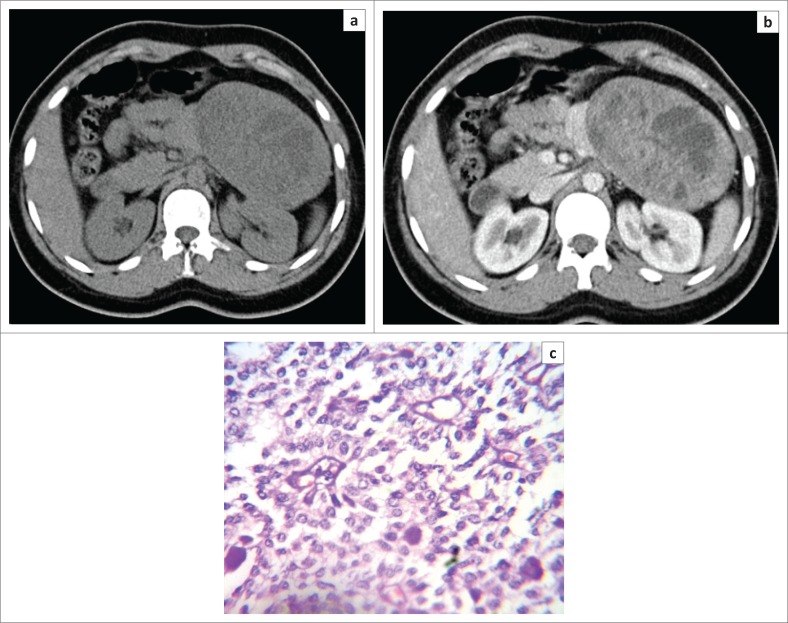
Axial non-contrast-enhanced computed tomography image (a) in a 31-year-old female patient showing a large lesion in relation to the tail of pancreas with a central hypodense area. Axial contrast-enhanced computed tomography image (b) in the same patient showing enhancement of the solid component of the lesion with non-enhancing areas suggestive of a solid pseudo-papillary tumour. High power magnification (40X) photomicrograph (c) of the same patient showing hyaline globules, pseudo-papillae and nuclear grooves confirming the diagnosis of solid pseudo-papillary tumour.

Solid pseudo-papillary tumour constituted 21.1% of pancreatic lesions in our study with 75% of patients under the age of 30 years, with a mean age of 26.5 years and was seen exclusively in females. It was predominantly located in head of pancreas (75%) (Figure 3) in our study, with only 25% cases in the tail region (Figure 4). These findings were in resonance with other studies.^33-37^ All the cases of SPT in our study had a solid cystic appearance on CT with an enhancing large solid component (Figures 3b and 4b). None had a pure cystic appearance. Casedei et al.^35^ reported four cases of solid pseudo-papillary neoplasms and all four were solid well-defined masses. Alves et al.^37^ conducted a study on 10 pancreatic solid pseudo-papillary neoplasms, and found that on radiology the tumour was solid cystic or solid with almost equal frequency. This disparity of results in the SPT appearance in our study could be accounted for because of exclusion of purely solid lesions. Kehagias et al.^38^ reported that enhancing solid areas in SPT are typically peripheral in location, whereas cystic spaces are usually more centrally located. Calcification was an inconsistent finding with variable pattern seen in only 2 two (50%) lesions in a peripheral location. Megibow et al.^21^ reported calcifications in 29% of SPTs. Common bile duct, MPD and the biliary tree were normal in all SPT cases consistent with the literature.^39^ No metastatic liver lesions were present in our study, although metastases can occur.^40^ Three of these lesions presumptively diagnosed as SPT on CT in our study turned out to be neuroendocrine tumours on HPE. We determined that non-functioning neuroendocrine tumours become large and undergo cystic change, thus closely resembling SPTs on cross-sectional imaging. Choi et al^41^ reported that SPTs with a minimal cystic component or no intra-tumoural haemorrhage are difficult to differentiate from islet cell tumours.

**FIGURE 5:**
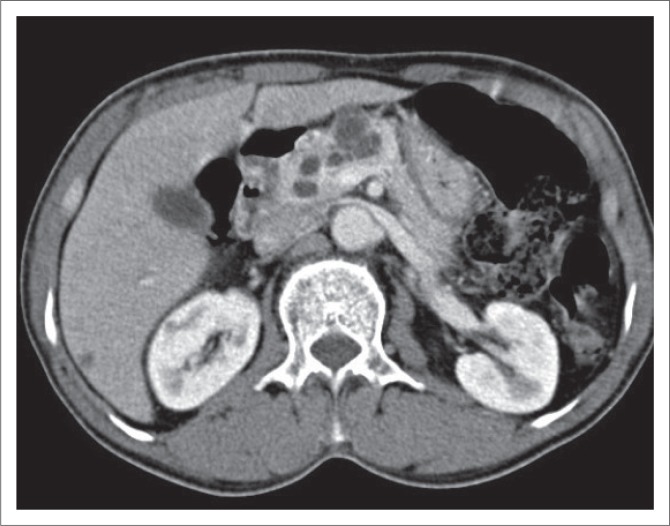
Axial contrast-enhanced computed tomography image in a 52-year-old male patient revealing a cystic lesion with septations in the body of pancreas with a dilated main pancreatic duct. A presumptive diagnosis of intra-ductal papillary mucinous neoplasm was made and histopathology examination confirmed the computed tomography diagnosis.

Intra-ductal papillary mucinous neoplasm was seen in 10.5% of our patients with a mean age of 56 years at the time of diagnosis and exclusively with a male distribution, consistent with the international literature reflecting a predominance in middle to elderly men.^42^ An equal number of lesions were seen in the head and the body region (Figure 5). Paal et al.^42^ reported that 18 of 22 IPMNs were present in the head. The mean size of the lesions at sonography was 2.65 cm, and at CT and pathology it was 2.5 cm, which is consistent with the literature.^43^ All the IPMNs appeared as cysts with internal septa (Figure 5). The main pancreatic duct was dilated in all the four cases (Figure 5); however, communication of the cyst with the MPD was difficult to identify in three patients and was identified only in one patient with certainty. Procacci^44^ found that at US and CT, branch duct tumours, which were mainly located at the uncinate process, were seen as fluid-filled masses with central septa and the pancreatic duct was dilated. Paal et al.^42^ conducted a study on 22 pancreatic IPMNs. Radiologically, the cases presented with inhomogeneous solid or cystic masses. However, a dilated pancreatic duct was present in all cases. We found that cystic pancreatic lesions with enhancing septa and dilated MPD in an elderly male are helpful pointers towards IPMN.

**FIGURE 6:**
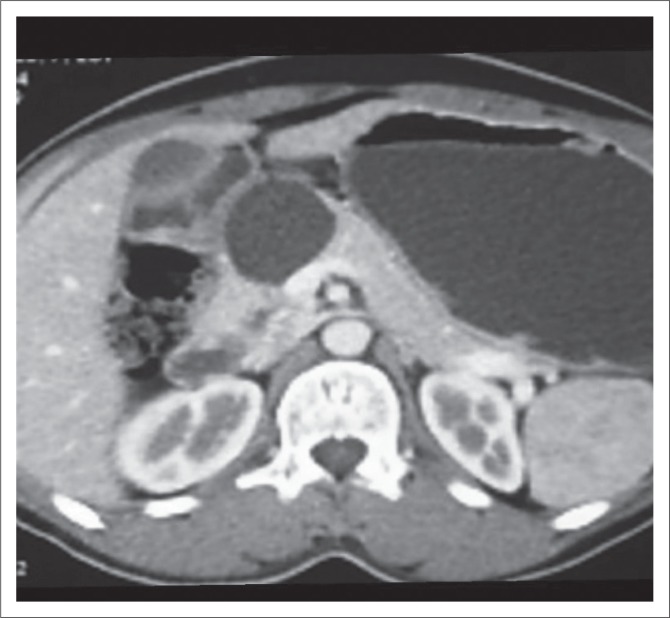
Axial contrast-enhanced computed tomography image in a 25-year-old male patient showing a simple pancreatic cyst. No septation or wall enhancement seen. Main pancreatic duct is not dilated.

Simple pancreatic cysts constituted 7.9% of the pancreatic cystic lesions with fluid attenuation on CT, and no septations, solid component or calcifications were seen within them. The outline was smooth. No enhancement was seen on post-contrast CT (Figure 6). The main pancreatic duct and biliary tree were not dilated. These features are consistent with the world literature.^45,46^ The least common cystic pancreatic lesion was pancreatic lymphangioma, with only one case seen in our study. The 55-year-old woman showed a multi-cystic peripancreatic lesion closely abutting the pancreas and insinuating in between the surrounding structures with no mass effect. Few tiny calcific foci were also seen; however, no definite fat attenuation was noted. A presumptive diagnosis of pancreatic lymphangioma was made on CT and subsequently proved on HPE.

Summarising, the overall diagnostic accuracy of CECT in diagnosing pancreatic cystic lesions was 72.5% in our study. Johnson et al.^22^ were able to definitively diagnose 93% of serous tumours (14/15 tumours), and Procacci et al.^47^ reported that CT findings allowed correct characterisation of only 60% of cystic pancreatic masses.

## Conclusion

The diagnostic accuracy of CT was high in case of SCA, IPMN and pancreatic cysts, and low in case of MCA and SPT. Combination of a multiloculated cystic lesion with locule size of less than 20 mm, septal enhancement with relative lack of wall enhancement, central scar and lobulated outline are highly specific for SCA. Unilocular or macro-cystic pattern with locule size of more than 20 mm, female gender and wall enhancement with smooth external contour are pointers towards MCA. Solid cystic pancreatic head lesions in young females may be suggestive of SPT. Dilated MPD in a cystic lesion with internal septations may point towards IPMN. Fluid attenuation lesions with imperceptible non-enhancing wall indicate pancreatic cysts. Lastly, pseudocysts and neuroendocrine tumours with a cystic component are great mimickers of pancreatic cystic lesions, and history of pancreatitis and hormonal profile of patients should always be sought.
